# Postictal psychosis in a psychiatric pacient, about a case

**DOI:** 10.1192/j.eurpsy.2023.1283

**Published:** 2023-07-19

**Authors:** M. V. López Rodrigo, M. Palomo Monge, A. Osca Oliver, V. Ros Fons, F. Tascón Guerra

**Affiliations:** Psiquiatría, Hospital Nuestra Señora del Prado, Talavera de la Reina, Spain

## Abstract

**Introduction:**

Postictal psychosis is the most frequent psychosis in epileptic patients, appearing between 3-8% of them. As temporary criteria, it must appear in less than a week after the epileptic crisis, with a duration of between 15 hours and 3 months.

**Objectives:**

We present the case of an 82-year-old patient admitted to the ED due to an epileptic seizure. Request evaluation for agitation and disorientation.

**Methods:**

This is an 82-year-old patient diagnosed with epilepsy for 5 years, with unwitnessed seizures, under treatment with levetirazetam. In the last consultation with neurology, the differential diagnosis was raised with anxiety crises due to normal intercritical EEG.The patient went to the emergency room after a partial crisis, presenting a post-critical state and beginning hours later with disorientation in time and place, manifesting delusions of religious content, as well as visual hallucinations of the same type, presenting agitation that required pharmacological and mechanical restraint.

**Results:**

Admission to the Neurology Service was decided, with a good response to treatment with a typical intramuscular antipsychotic, with complete remission of the condition in 48 hours. Small areas of ischemia compatible with the patient’s age are observed in the cranial CT and the EEG shows slowed global activity.

**Conclusions:**

Postictal psychosis is a phenomenon of low prevalence, however, it is important to take it into account. It is important to recognize the postictal “lucid” period in patients with a family or personal history of psychiatric illness and seizures with compromised consciousness.

**Disclosure of Interest:**

None Declared

